# Elasticity, an often-overseen parameter in the development of nanoscale drug delivery systems

**DOI:** 10.3762/bjnano.14.95

**Published:** 2023-11-23

**Authors:** Agnes-Valencia Weiss, Marc Schneider

**Affiliations:** 1 Department of Pharmacy, Biopharmaceutics and Pharmaceutical Technology, Saarland University, Campus C4 1, Saarbruecken, Germany

**Keywords:** atomic force microscopy, drug delivery, elasticity, mechanical properties, nanomedicine, nanoparticles, stiffness measurement, tissue/body distribution

## Abstract

Nanoparticles have shown an enormous potential as drug delivery systems in the lab. However, translation to the clinics or even market approval often fails. So far, the reason for this discrepancy is manifold. Physicochemical properties such as size, surface potential, and surface chemistry are in focus of research for many years. Other equally important parameters, influencing whether a successful drug delivery can be achieved, are mechanical properties of nanoparticles. Even though this is often not even considered during formulation development, and it is not requested for approval, an increasing number of studies show that it is important to have knowledge about these characteristics. In this article, we discuss examples highlighting the influence of elasticity in nanoscale biological interactions focusing on mucosal delivery and on tumor targeting. Besides this, we discuss the influence of different measurement settings using atomic force microscopy for the determination of mechanical properties of drug carriers.

## Introduction

Drug delivery systems are developed with the aim to transport a given drug to the site of action followed by the release of the drug. Therefore, three major benefits are expected when tailoring those systems: to overcome barriers, which would hinder the drug to reach the side of action, to decrease side effects by less unspecific drug action in nontarget areas, and to lower the overall dose to be encapsulated obtaining therapeutic relevant concentrations [1–2]. Nanoparticulate drug delivery systems have been researched for more than 35 years and show promising outcomes in the lab [3–4]. Unfortunately, even though nanoparticulate formulations often deliver positive results in vitro, the translation to in vivo and even more to the clinics often fails and only a limited number of products make it to the market. This holds true even though Comirnaty^®^ and Spikevax^®^ were approved during the SARS CoV-2 pandemic using lipid nanoparticle (NPs) formulations underlining their potential [5–7]. By examining the characteristics of nanoparticles used for drug delivery, one can see that some are better understood then others. The size of nanoparticles, for example, is shown to play an important role in tissue or mucus penetration [8] and in cellular uptake [9]. Also surface charge and chemical properties are well investigated regarding their effect on cellular uptake and the influence on in vivo performance [3,10]. Only since the first decade of the 2000s, mechanical properties have been investigated in the development of nanoparticulate drug delivery systems. A well-known example in biology, demonstrating the impact of these characteristics, is the life span of red blood cells. Juvenile red blood cells are able to flow through capillaries much smaller in diameter than their size due to sufficient elasticity. During their life span, they gain rigidity leading to their filtration out of the blood system when they reach the end of their lifetime [11–12]. Similar data were reported for nanoparticulate systems [13]. Other examples are viruses [14] and cancer cells which can adapt their mechanical properties multiple times during the process of metastasis formation [15]. Looking at these examples, one can appreciate the relevant role of mechanical properties of biological systems. However, it is surprising that mechanical properties still play a minor role in the development of drug delivery systems.

Even though there is an increasing number of studies focusing on the influence of mechanical properties of nanoparticles, it nearly impossible to compare studies from different labs and pick a value to aim for during the development of a drug delivery system. This is due to the variety of possibilities to examine and express the mechanical properties of these systems (e.g., stiffness, elasticity determined by the Young’s modulus, bulk modulus or shear modulus, viscoelastic properties or deformability) as well as the measurement method to quantify these properties. Anselmo et al. as well as Nie et al. gave comprehensive overviews and definitions of different measurements of mechanical properties [16–17]. Similarly to the advent of nanomedicines, certain standardization in terms of methods and parameters is necessary to allow for a better comparison between different studies.

## Perspective

### Measurement conditions influencing the absolute values of elastic properties

Different possibilities to determine mechanical properties of nanoparticles (or their corresponding bulk materials) highlighting quartz crystal microbalance, rheology, and atomic force microscopy (AFM) are summarized by Li et al. [18]. Another often reported method is particle deformability, being extrusion a possibility for nanoparticles [13] and microfluidic setups for particles that are large enough to be imaged by light microscopy techniques [19]. As AFM is currently the only technology capable to measure mechanical properties of single nanoparticles, we will concentrate on this technique.

**Figure 1:**
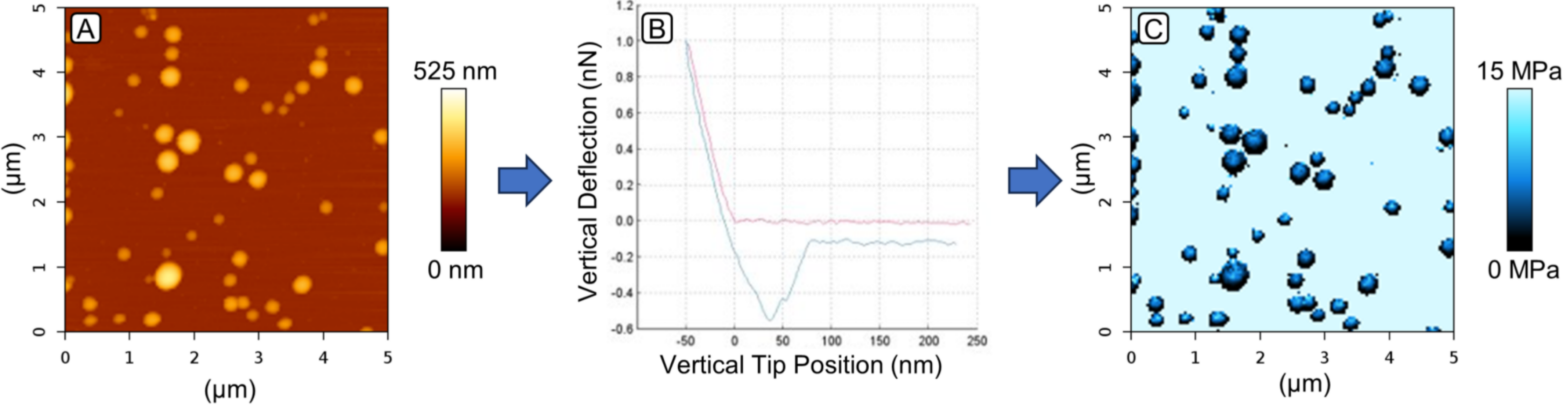
(A) Height image of gelatin nanoparticles on a silica surface imaged in Milli-Q^®^ water at 37 °C in the quantitative imaging mode. (B) A force–distance curve from a pixel representing the middle of a particle, and (C) the corresponding Young’s moduli map. The elasticity is often extracted from the slope of the approach curve right after the contact point with the surface (B).

Atomic force microscopy currently provides different possibilities of measuring forces at the nanoscale. This can be the acquisition of single force–distance curves on a specific spot after locating the particle in an imaging mode or the creation of whole maps using quantitative imaging modes [20–22]. An example is shown for lysozyme-loaded gelatin nanoparticles imaged in the quantitative imaging mode with a JPK NanoWizard III in Milli-Q^®^ water at 37 °C, as well as the extracted Young’s modulus map as previously described [22] (Figure 1). Takechi-Haraya et al. showed that for liposomes both methods deliver the same results [21].

The most popular method is the evaluation of nanoindentation data resulting in Young’s moduli. The determination of the Young’s modulus is based on different theories. The adapted Hertz’ model, according to Sneddon [23], is most often used requiring a maximum indentation of 10% of the particle height. However, more complete approaches can be applied [24], which consider the situation of a given system. An example is the Johnson–Kendall–Roberts (JKR) theory, including surface forces, the influence on the adhesion, deformation, and contact behavior between the nanoparticle and the AFM probe suitable for soft and deformable objects [25]. Dealing with hard materials might require to apply the Derjaguin–Muller–Toporov (DMT) theory [26]. Garcia gives a comprehensive overview about different models and contact theories for mechanical measurements of soft materials using AFM [27].

**Figure 2:**
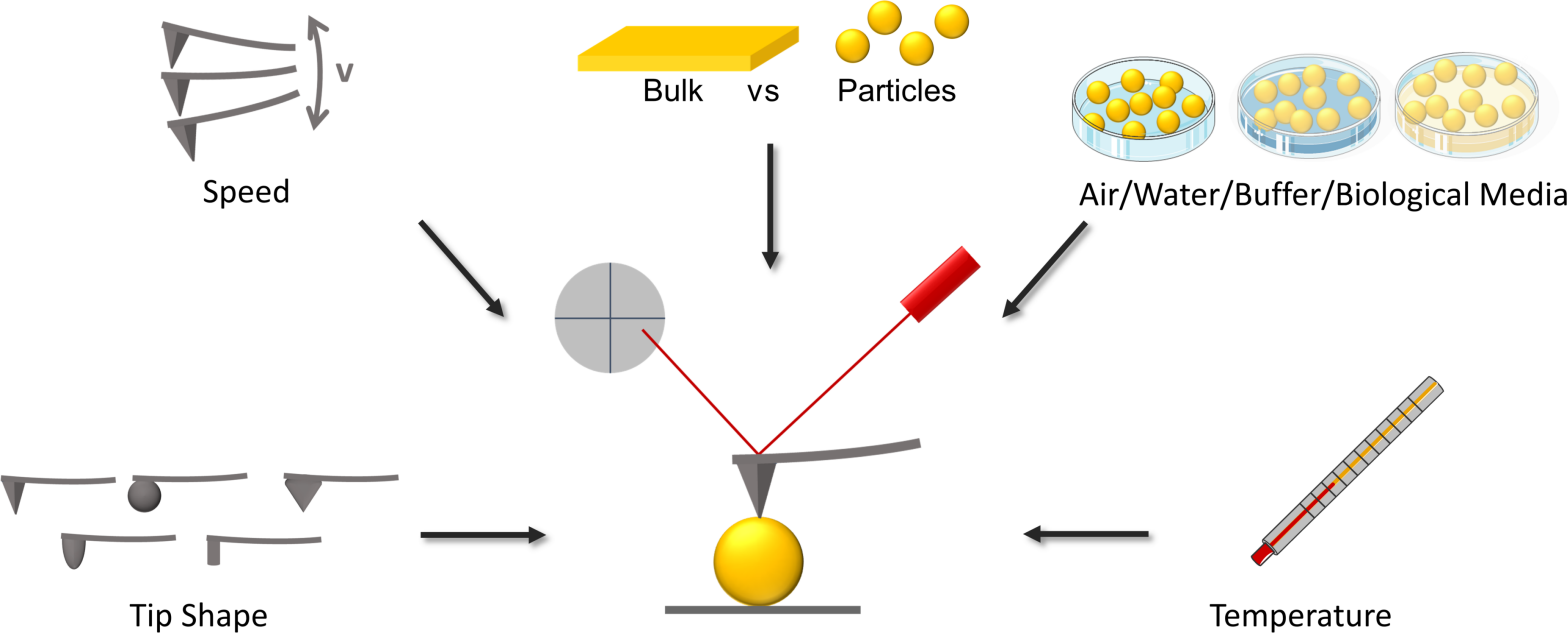
Factors influencing the absolute values of measured elasticity of nanoparticles determined by AFM. These factors mainly are tip shape, acquisition speed, measurement on bulk material or directly on nanoparticles, elasticity determination in air, water, or buffers, and the temperature. Parts of this figure were created by using pictures from Servier Medical Art provided by Servier, licensed under a Creative Commons Attribution 3.0 Unported License (https://creativecommons.org/licenses/by/3.0/). This content is not subject to CC BY 4.0.

Further options are the implementation of pause segments keeping the force or position constant and monitoring the other variable. This opens the possibility to measure viscoelastic properties determining, in the case of the Zener model, the elastic modules (*E*_0_, *E*_1_) and the viscosity (η). More recently, the storage modulus *E*' and the loss modulus *E*'' have been obtained by performing oscillatory measurements in contact with the sample. If maps are created and the recorded data is evaluated via batch processing, these moduli can be displayed as images and correlated with height images. The best option to determine mechanical properties of single nanoparticles might be dependent on the studied material, as some materials show viscoelastic properties whereas others might show pure elasticity. However, if one is aiming for a more standardized method to characterize mechanical properties of nanoparticles in a way that particle sizes or surface potentials are determined, we would suggest to prioritize the determination of the Young’s modulus. This is due to the advantage of comparable fast acquisition and the mapping option. From our own data on gelatin nanoparticles, we can say that introducing pause segments during the measurement with constant force or constant height, when in contact with the particles, the information might be more precise as we get viscoelastic information and not only elasticity. However, the acquisition as well as the evaluation of these measurements on nanoparticles is much more time consuming as well as prone to introducing errors. Overall, the tendency in the results is the same as for the elasticity-based determination of the Young’s modulus. However, not only differences in the measurement methods but also differences in the nanoparticle properties makes it hard to directly compare different studies. Even when only looking into elasticity studies performed by AFM, it is nearly impossible to compare the absolute values as the measurement conditions and settings can significantly influence the obtained results. Although much more challenging, it is of outmost importance to perform measurements on nanoparticles and not only on bulk materials [28]. A corresponding bulk material gives significantly different elasticity values than those of the actual NPs even if using the same measurement conditions. This was already demonstrated by Alsharif et al. [29]. Furthermore, they could show the high impact of performing the measurement in air or in water, resulting in lower values using water due to swelling and/or softening effects. The knowledge about the influence of measurement conditions on the absolute elastic value is still limited. Temperature is an important factor in the context of elasticity of nanomaterials. Even though the exact temperature of the particles is not known, it is assumed to be equal to the surrounding temperature after a sufficient equilibration time. Many studies are performed at room temperature or at 25 °C instead of at the temperature in which biologic studies are performed. In some cases, the temperature is not specified [30–32]. At least for the polymeric particles studied by Alsharif et al., this makes a significant difference as it is expected for all particles to swell or interact with the medium. For the determination of cellular elasticity, several measurement parameters are well studied. Indentation speed, applied force, and tip shape are some examples. Faster indentation leads to enhanced Young’s moduli [33–34] and rounded tips result in lower Young’s moduli compared to those of sharp pyramidal or quadratic pyramidal tips. Additionally, larger tip radii lead to lower elastic values [33,35]. Figure 2 summarizes factors that impact the elasticity measurement results obtained by AFM.

Due to the issues aforementioned discussed, it is tricky to compare different studies. In addition, it is not yet known which absolute elastic values should be chosen during formulation development. There is a clear trend for biological interactions among particles of the same material. However, different mechanical properties are expected when comparing across these particles. This topic will be discussed in the subsequent section.

### Nanoparticle elasticity and biological applications

Mechanical properties of particles have a significant impact on the cell–particle interaction; most particles are reported to be taken up faster when they are more rigid [36]. Some materials such as phospholipids and organic silica NPs with a hyaluronic acid coating show superior uptake for softer particles [30]. Cell uptake is often the first biological evaluation during the development phase besides toxicity and biocompatibility. However, after application, particles first need to reach the cells and overcome several other biological barriers. During uptake, other biological barriers besides cellular membranes need to be addressed. A few examples of these barriers are penetration in or permeation through mucus, skin penetration, overcoming the blood brain barrier, or extravasation from blood vessels. Another challenge is the accumulation of particulate drug delivery systems in certain tissues. There are not many studies available to address the influence of particle elasticity on the interaction with these barriers. However, there is a clear trend that shows that softer particles seem to be beneficial. In the following section we will highlight some of the routes.

### Tuning particle elasticity to overcome the mucosal barrier

Mucus covers a large area of our body and is an important barrier for many drugs as it covers common application routes such as the intestines, the lungs, nose, and vagina. Regarding the penetration through mucus, Lenzini et al. demonstrated in a study with a model hydrogel that there is a higher penetration for more deformable extracellular vesicles from mouse mesenchymal stromal cells [37]. A second study, from Yu et al., shows rigidity-dependent penetration of lipid NPs in the mucus layer of rat intestinal mucus. Liposomes were either hollow or filled with poly(lactic-*co*-glycolic acid) (PLGA) cores of different sizes resulting in interfacial water layers with different thicknesses and therefore with tunable elasticity [38]. Semielastic particles whose Young’s moduli were around 50 mPa showed the fastest diffusion in mucus. However, harder particles showed better cellular uptake if no mucus layer was present. In contrast to this, the cellular uptake for semielastic particles was not significantly affected by the presence of a mucus layer [38]. Liposomes with PLGA cores were used by Yu et al. to increase the stiffness in combination with surface modification, leading to a prolonged pulmonary retention of dexamethasone-loaded nanoparticulate drug carriers for the treatment of acute pulmonary inflammation [39]. This is in accordance with a study from Zheng et al. where crosslinked insulin-loaded hydrogel zwitterionic nanoparticles with variable elasticity were prepared by immersing mesoporous silica nanoparticles in carboxybetaine methacrylate (CBMA) and crosslinking those with different amounts of carboxybetaine dimethacrylate (CBMAX). Particles with Young’s moduli between 4.5 and 162 MPa (measured in air) were obtained. By measuring the interaction with mucus in vitro, they demonstrated that the apparent permeability coefficient (Papp value) through porcine intestinal mucus as well as the diffusion determined by particle tracking is significantly higher for softer particles. However, as soon as cells are included in the system (e.g., Caco-2 monolayers, or HT29-MTX-E12 cells, or an in vitro model of a rat everted intestinal sac) the Papp values are higher for NPs with higher Young’s moduli [40]. The blood glucose levels of diabetic rats treated by gavage with insulin or insulin-loaded NPs of different Young’s moduli were measured. The results show that the plasma insulin levels where higher for harder NPs, and this correlated with blood glucose reduction. Furthermore, 3 h after application, they found a larger amount of hard NPs in the intestinal villi [40].

All studies support our hypothesis that mechanical properties of nanoparticulate drug delivery systems are an important characteristic, and more attention should be paid to that during formulation development. Mechanical properties need to be fine-tuned for the intended target and according to therapeutic needs. Furthermore, the results demonstrate that in vitro assays need to be carefully chosen to be able to deliver a realistic outcome, facilitating translation of nanoparticles to in vivo applications (e.g., the presence of mucus altering the preferable mechanical properties). Besides this, the correlation between size and elasticity should also be considered as an important parameter.

### Tissue distribution and blood circulation

Independent of the application route, blood circulation and tissue distribution are important factors regarding the fate of nanoparticles in vivo. The plasma half-life time is not only important for a sustained release but also to enhance the time for passive and active drug targeting before particles are filtered out of the circulation system. Overall, it was shown that softer particles have an enhanced blood circulation time. The effects described were observed for different particle types (made from different materials) and also covered different ranges of Young’s moduli. The information is summarized in Table 1. The observed uptake and interaction behavior covered a broad range from 10 kPa to 4.7 GPa. Since different materials and architectures were used to prepare the particles, it might be concluded that the elasticity parameter is overruling material properties.

**Table 1:** Overview of mechanical properties of nanoparticles addressed in this article as well as particle-forming materials, conditions for the elasticity determination, and the influence on their biological behavior.

Particle material	Mechanical properties	Measurement conditions	Influence on the biological behavior	Ref.

poly(carboxybetaine) (pCB) loaded with gold NPs	bulk modulus: 0.18–1.35 MPa	n.a.*	softer NPs show longer blood circulation and lower accumulation in the spleen	[13]
liposomes (distearoylphosphatidylcholine (DSPC), distearoyl phosphatidylglycerol (DSPG), cholesterol; 53/21/26, (mol % ratio)	stiffness: 32 pN/nm	aqueous glucose solution (isotonic, pH 5.3) at 25 ± 1 °C	no biological evaluation	[21]
gelatin B, bloom ≈75 g	Young’s modulus: 1.06–14.26 MPa, (4.12–9.8 MPa used for biological studies)	quantitative mapping by AFM, Milli-Q^®^ water, 37 °C	stiffer GNPs are taken up faster and to a greater extend	[22,36]
poly(ethylene glycol) diacrylate (PEGDA)	bulk modulus 10–3000 kPa (softer NPs not used for biological studies)	room temperature, AR-G2 rheometer, parallel plate geometry	softer NPs have a longer blood circulation time and are taken up to a lower extend by endothelial and epithelial cells	[28]
PLGA and PLA of different molecular weights	Young’s modulus: 0.83–4.7 GPa	comparison between bulk, NPs in air versus in water, and at 25 versus 37 °C	no biological evaluation	[29]
hyaluronic acid modified mesoporous organosilica nanoparticles	Young’s modulus: 0.29–1.64 GPa	Young’s modulus, AFM, imaging in water, force measurements: n.a., 25 °C	softer NPs show enhanced cell uptake, prolonged circulation time, and higher tumor accumulation	[30]
poly(ethylene glycol) diacrylate (PEGDA, MW 600)	Young’s modulus: 0.37–3.15 MPa	quantitative mechanical mapping, temperature: n.a. liquid/air: n.a.	softer NPs with higher tumor targeting potential. Cell uptake at static conditions higher for stiffer particles and, at flow conditions, higher for softer particles with RGD modification	[31]
1,2-dioleoyl-sn-glycero-3-phosphocholine (DOPC) liposomes hollow or with an alginate core	Young’s modulus: 45–19,000 kPa. Particles for biological studies: <1.6 MPa and >13.8 MPa	force measurements, AFM, in water, temperature: n.a.	softer particles exhibit higher cell uptake and have an enhanced tumor targeting effect	[32]
extracellular vesicles with/without aquaporins	Young’s modulus: ~75–210 MPa	force–displacement curves, AFM, temperature: n.a. air/liquid: n.a.	higher vesicle deformability (softer vesicles) results in higher hydrogel penetration	[37]
phosphatidylcholine, cholesterol and Pluronic F127 (1/28/5, molar ratio) liposomes hollow or filled with PLGA cores	Youngs’s modulus: 5–110 MPa	PeakForce QNM imaging mode, AFM, room temperature, 85% humidity	semisoft particles show best mucus diffusion, cell uptake with overlaying mucus layer and highest plasma levels after oral administration	[38]
phospholipid, cholesterol, and DSPE-PEG2000 liposomes hollow or filled with PLGA cores	shear modulus: 84–2020 kPa	quartz crystal microbalance (QCM), 37 °C, water	stiffer particles show enhanced pulmonary retention as well as higher endo- and and exocytosis	[39]
carboxybetaine methacrylate (CBMA) & carboxybetaine dimethacrylate (CBMAX)	Young’s modulus 4.46–165.2 MPa	force–displacement curve, AFM, in air, room temperature	stiffer particles exhibit lower mucus penetration but enhanced epithelial transcytosis	[40]

*n.a.: not available.

The tissue distribution can be important regarding active and passive targeting of different tissues, such as tumors or inflammation sites. It also gives an idea about possible side effects as high nanoparticle concentration usually correlates with high drug concentration. Nanoparticles often show high accumulation in the liver, where particles are cleared by the reticuloendothelial system (RES), and in the spleen due to its filtering function. Softer hydrogel nanoparticles composed of poly(carboxybetaine) [13] as well as poly(ethylene glycol) diacrylate (PEGDA) [28] showed longer blood circulation times. Anselmo et al. demonstrated that their softer PEGDA particles are found at a lower amount in the liver compared to stiff particles of the same material [28]. This is an important aspect regarding the potential side effects of the nanoparticles as most of the pharmaceutically applied nanoparticles is accumulated in the liver.

The significance of elastic properties of nanoparticles regarding the passive tumor targeting is addressed in the next section.

### Soft particles for enhanced passive tumor targeting

A comprehensive review about the mechanisms involved in tumor targeting, how elasticity contributes to an enhanced tumor targeting, as well as strategies to alter mechanical properties of nanoparticulate drug delivery systems is given by Hui et al. [41]. In this perspective, we concentrate on giving and discussing some literature examples. Guo et al. showed the enhanced tumor accumulation and deeper penetration of nanolipogels (NLGs) [32]. The particles had a phospholipid double layer and except for the softest formulation, their cores were filled with agarose gels of different gel strength values. With this method they were able to prepare nanoparticles with comparable size and surface characteristics but variable Young’s moduli ranging from 45 kPa to 19 MPa. In contrast to many other studies, soft nanoliposomes (NLPs) are taken up better by cells than harder particles used in this study. This is explained by the combination of membrane fusion and clathrin-mediated uptake mechanisms, whereas hard NLGs are predominantly internalized via the clathrin-mediated pathway [32]. Another example of the enhanced tumor accumulation of softer nanoparticles is presented by Tao et al. Even though the hyaluronic-acid-modified mesoporous organosilica nanoparticles (MMONs) they investigated were much stiffer (Young’s Moduli between 0.29 and 1.64 GPa, at 25 °C), the tendency was the same as reported from other groups: softer particles accumulate to a higher extend in the tumor tissue [30]. Interestingly, in their study, softer particles were taken up to a larger amount into the tumor cells with the highest uptake for particles with a Young’s modulus of 0.4 GPa. This trend is the opposite to the majority of other studies and could have several reasons. On the one hand, the hyaluronic acid might modify the particle–cell interaction and foster uptake. On the other hand, the high absolute Young’s moduli in the upper MPa up to the GPa range could also be a reason for the observed effect. However, the elasticity in the in vitro experiments might be different as particles in this study were investigated at 25 °C instead of at 37 °C. Additionally, from the methods described in the supporting information, it is not clear in which surrounding medium they performed the measurements leaving an uncertainty about the mechanical properties of the particles during the biological studies. A third reason could be the changed morphology of softer MMONs as the treatment results in raisin-shaped NPs and therefore soft MMONs show locally small curvatures, which might facilitate cellular uptake.

An interesting approach using elasticity as a tumor targeting factor is presented by Chen et al. In their study they prepared hydrogel nanoparticles (HNPs) based on poly(ethylene glycol) diacrylate (PEGDA) with Young’s moduli between 0.37 and 3.15 MPa. Unfortunately, the authors do not state the elasticity measurement conditions. Additionally to the passive tumor targeting by elasticity, the particle surface was modified with cyclic arginyl-glycyl-aspartic acid (RGD) as a tumor-targeting molecule to achieve an active receptor-mediated cell uptake. The receptor binding results in an enhanced adhesion to cells which is further increased by the deformability of the particles. This results in an enhanced cellular uptake in both static and flow conditions. This is described to be due to a larger contact area between NPs and cells resulting in enhanced RGD interaction with the integrins on the cell surface [31]. For cells lacking the RGD receptor, such as the macrophage cell line RAW264.7, the cellular uptake of soft particles is significantly lower than that of stiff particles. HeLa cells show an enhanced uptake by RGD functionalization but it is still reduced in comparison to all hard formulations. Particles with RGD-modified surfaces are superior in tumor accumulation for both soft and hard HNPs, with softer particles showing an overall higher retention in the tumor. This results in the highest particle accumulation in the tumor tissue for RGD surface-modified soft hydrogel nanoparticles. Unfortunately, in the study of Chen et al. the particles performing best in tumor delivery are found with the highest concentration in the spleen and in the liver where their cargo can potentially cause undesired side effects [31]. Nevertheless, effective cellular uptake of the majority of soft nanoparticles can be improved either by tuning the material properties or by active targeting. The usage of nanoparticles with high deformability for enhanced passive tumor targeting seems to be a very promising formulation parameter, which is worth to be further explored in future studies.

## Conclusion and Future Directions

Despite all challenges regarding influencing and measuring mechanical properties of nanoparticles for drug delivery, we are convinced that elasticity is a parameter that should be addressed during the development of nanoparticulate drug delivery systems and stability tests. Materials specific interaction and elasticity might strongly contribute to the variable outcomes of nanomedicinal approaches and limited translation to the clinics. Cooperation between experts who develop methods and devices and formulation scientists could improve standard characterization techniques, such as size or surface potential, which are already given in almost every study involving nanoparticles. A full characterization with respect to size, surface potential, corona formation on the surface, and elastic properties should be done. In a best-case scenario, certain conditions would always be set to a certain range to facilitate comparison and guarantee relevant settings. However, some parameters clearly influence the measurement results and thus should be the minimum reported parameters in publications. This is also recommend for other nanoparticulate properties and characterization techniques as well as biological experiments with the aim of harmonization of research data [42–43]. However, more efforts are needed to establish a standardized determination of mechanical properties. Furthermore, it should be common that measurement conditions such as temperature, applied force, and indentation speed are provided. Also, if measurements are performed in water or in buffer, the composition needs to be given in order to achieve better comparability. In Table 2, the parameters that need to be considered during the experiment planning and which contribute to the measurement results are summarized. These settings should also be reported in manuscripts for comparison across different studies.

**Table 2:** Parameters and measurement settings during the determination of Young’s moduli, which should be reported in publications.

Measurement settings which should be defined in publications and considered during experiment planning	

AFM settings	measurement mode, acquisition speed, applied force
cantilever	tip shape, spring constant and resonance frequency of the cantilever, calibration method, applied correction factor during calibration
measurement conditions	temperature, air or liquid surrounding conditions including type of liquid (e.g., medium, water, concentration and type of salts used)
Young’s modulus extraction	Hertz/Sneddon model, JKR model, DMT model
particle location	at which point of the object is the elasticity determined? size selection? size and indentation of the tip

Even though the in vivo fate of particles is very complex and depends on many particle characteristics, the currently available studies underline that the elasticity of drug delivery systems has a significant impact. Due to this, more effort is needed to understand how elasticity must be tuned for different application routes and targets leading to highly efficient drug delivery. In addition, to investigate biological effects the cell type (i.e., epithelial, immune, or even more organ-specific cells) should be monitored and well documented. This will hopefully allow for the separation and better understanding of the obtained results, such as biodistribution, tissue accumulation, and cellular uptake. We are convinced that elasticity as a formulation parameter can help to promote more nanoparticulate drug delivery systems to be translated to the clinics.
